# A Masked, Randomized, Phase 3 Comparison of Triple Fixed-Combination Bimatoprost/Brimonidine/Timolol versus Fixed-Combination Brimonidine/Timolol for Lowering Intraocular Pressure

**DOI:** 10.1155/2017/4586763

**Published:** 2017-09-19

**Authors:** Curt Hartleben, Juan Camilo Parra, Amy Batoosingh, Paula Bernstein, Margot Goodkin

**Affiliations:** ^1^Instituto de Oftalmología Conde de Valenciana, Chimalpopoca 14 Colonia Obrera, Deleg. Cuauhtémoc, 06800 México City, Mexico; ^2^Fundación Oftalmológica de Santander, Medical Center Carlos Ardila Lulle, Tower A office 401 11 module, Bucaramanga, Colombia; ^3^Allergan plc, 2525 Dupont Drive, Irvine, CA, USA

## Abstract

**Objective:**

To evaluate the efficacy and safety of triple fixed-combination bimatoprost 0.01%/brimonidine 0.15%/timolol 0.5% (TFC) versus dual fixed-combination brimonidine 0.2%/timolol 0.5% (DFC) in primary open-angle glaucoma and ocular hypertension.

**Methods:**

Patients with intraocular pressure (IOP) ≥23 and ≤34 mmHg were randomized to twice-daily TFC or DFC. The primary variable is the change in worse eye mean IOP from baseline at week 12 (modified intent-to-treat (mITT) population). Secondary endpoints are mean IOP and mean change from baseline at weeks 1, 2, 4, 8, and 12 (mITT population). TFC superiority was demonstrated if the primary variable favored TFC (*p* ≤ 0.05). Sensitivity analyses were conducted, and safety was assessed at all visits.

**Results:**

TFC (*n* = 93) provided greater IOP reductions from baseline than DFC (*n* = 97) at week 12 (treatment difference, 0.85 mmHg; *p* = 0.028) and all other visits. TFC was also superior to DFC in patients with high baseline IOP (i.e., IOP ≥ 25 mmHg; *p* ≤ 0.011). Conjunctival hyperemia, ocular irritation, and dry eye were reported more often with TFC (*p* ≤ 0.016); however, discontinuations for ocular adverse events were similar between treatments.

**Conclusions:**

TFC demonstrated IOP-lowering benefits that outweigh the risk of predominantly mild ocular side effects, which may be particularly relevant in patients who require greater IOP lowering to prevent/delay disease progression. This trial is registered with ClinicalTrials.gov registry number: NCT01241240.

## 1. Introduction

Elevated intraocular pressure (IOP) is a key risk factor for the development and progression of glaucomatous optic neuropathy, and the only factor that has been modified therapeutically to date [1–4]. In the Early Manifest Glaucoma Trial (EMGT), a correlation was observed between the magnitude of the IOP decrease and disease progression, with each 1 mmHg decrease in IOP reducing the risk of progression by an estimated 10% [5]. Accordingly, topical hypotensive drugs that reduce IOP by inhibiting aqueous humor production and/or increasing outflow constitute the mainstay of pharmacological therapy to prevent visual loss in patients with glaucoma or ocular hypertension (OHT) [1, 6–8].

The *β*-blocker timolol has a long history of use as monotherapy for primary open-angle glaucoma (POAG) and OHT [9]. Prostaglandin analogs/prostamides such as bimatoprost were first approved in 1996 [10] but have become increasingly preferred as first-line options due to their efficacy, safety, and once-daily use [2, 10–14]. The *α*2-adrenergic receptor agonist brimonidine is usually administered two or three times daily and provides the potential therapeutic advantage of a dual mechanism of action, inhibiting production of aqueous humor (like timolol) and stimulating uveoscleral outflow (like bimatoprost) [2, 15, 16]. Brimonidine is also thought to have neuroprotective effects, based on preclinical data [17–21] and indirect clinical evidence [22, 23]. Nonetheless, combinations of agents are often required to control IOP and prevent disease progression [1, 2]. The Ocular Hypertension Treatment Study (OHTS) indeed showed that at 60 months, 39.7% and 9.3% of patients treated for elevated IOP required at least two and three medications, respectively, to achieve their target IOP [24]. These proportions are clinically relevant considering that glaucoma requires life-long treatment.

Bimatoprost, brimonidine, and timolol are commonly used concurrently. Fixed combinations offer the advantages of limiting exposure to preservatives and reducing the occurrence and severity of hyperemia and other adverse events (AEs), minimizing drug washout due to consecutive instillations, lowering costs, and increasing adherence and persistence to treatment [1, 2, 15, 25–30]. Various clinical studies have demonstrated the tolerability and greater IOP-lowering efficacy of dual fixed-combination therapies such as once-daily bimatoprost 0.03%/timolol 0.5% (Ganfort®; Allergan plc, Irvine, CA, USA), twice-daily brimonidine 0.2%/timolol 0.5% (Combigan®; Allergan plc), and three-times-daily brinzolamide 1%/brimonidine 0.2% (Simbrinza®; Alcon Laboratories Inc., Fort Worth, TX, USA), compared with their individual components [31–42]. We thus hypothesized that in patients whose IOP is not controlled, treatment with a formulation containing three complementary agents may result in additional IOP lowering, compared with a dual-combination formulation, with no clinically relevant impact on tolerability. To test this hypothesis, the efficacy and safety of a new bimatoprost 0.01%/brimonidine 0.15%/timolol 0.5% ophthalmic solution (triple fixed-combination (TFC)) were evaluated in patients in Mexico and Colombia who had elevated IOP due to POAG or OHT, compared with brimonidine 0.2%/timolol 0.5% (dual fixed-combination (DFC), Combigan).

## 2. Methods

### 2.1. Study Design

This multicenter, double-masked, randomized, phase 3 study (ClinicalTrials.gov registry number: NCT01241240) was conducted in eight centers in Mexico and Colombia, in accordance with the guidelines of Good Clinical Practice and the International Conference on Harmonisation, as well as applicable local laws. The study was approved by an institutional review board at each investigational site (see Acknowledgments for a listing) prior to study start, and each patient provided written informed consent before initiating treatment.

### 2.2. Participants

The key inclusion criteria were ≥18 years of age; diagnosis of POAG or OHT requiring bilateral ocular hypotensive treatment; ability to undergo washout of prior IOP-lowering therapy (if applicable); baseline IOP ≥ 23 and ≤34 mmHg in both eyes; and best-corrected visual acuity ≥ 20/100 in each eye. The key exclusion criteria were presence of uncontrolled systemic disease; known history of nonresponse to previous bimatoprost treatment; known allergy/hypersensitivity to the study medications or their components; contraindication to brimonidine or *β*-blockers; presence of active or recurrent ocular disease other than POAG or OHT (except chronic mild blepharitis, cataract, age-related macular degeneration, or background diabetic retinopathy); required chronic use of other ocular medications during the study; functionally significant visual field loss or evidence of progression in the last year; recent (within 30 days before the screening visit) or anticipated alteration of existing chronic therapy with agents that could substantially affect IOP; conjunctival hyperemia > +1.0 (i.e., mild) or other active ocular surface findings at baseline; history of ocular disease and/or ophthalmic surgical or laser procedures that could confound study data or influence patient safety; and history of cataract surgery within 6 months of study start.

### 2.3. Treatment and Assessments

At the screening visit, patients using topical IOP-lowering therapy initiated a washout period of 4 days to 4 weeks (depending on the drug category). At the baseline visit (day 0), eligible patients were randomized to TFC or DFC (with stratification based on use [yes/no] of systemic *β*-blockers) and instructed to instill one drop of the assigned treatment at approximately 12-hour intervals (i.e., between 08:30 and 10:30 and between 20:30 and 22:30) in both eyes for 12 weeks (starting on the evening of day 0). On the day of a scheduled visit, the morning dose of study medication was to be administered at the study site following completion of all hour-0 (between 08:00 and 10:00) assessments.

Study treatments were supplied by Allergan in kits of identical appearance to maintain the double-masked nature of the study. Assessment visits were scheduled at baseline and weeks 1, 2, 4, 8, and 12, and investigators were instructed to examine each patient at approximately the same time of day at each visit. IOP was measured in each eye by Goldmann applanation tonometry using a masked two-person reading method; two consecutive measurements were performed at both hour 0 (between 08:00 and 10:00) and hour 2 (2 hours later), followed by a third measurement at each time point if the difference was >2 mmHg. For each patient, the worse eye was the eye with the higher IOP at baseline (hour 0) or the right eye if both had the same IOP. Mean IOP was the average of the worse eye IOP measurements at hours 0 and 2 of a given assessment visit.

### 2.4. Outcome Variables and Analyses

Primary and secondary efficacy analyses were performed in the modified intent-to-treat (mITT) population (defined as all randomized patients with at least one postbaseline efficacy evaluation) and repeated in the per-protocol (PP) population (defined as all randomized patients who received study medication, had no major protocol violations, and completed the treatment or were discontinued due to a lack of efficacy or AEs) for sensitivity analysis. All *p* values presented are 2-sided; analyses were generated using SAS® software version 9.3 (SAS Institute Inc., Cary, NC, USA).

The primary efficacy variable was the mean IOP change from baseline at week 12. Superiority of TFC over DFC was demonstrated if TFC showed a greater mean IOP reduction from baseline at week 12 than did DFC, with the resulting between-treatment difference *p* value ≤ 0.05. The last-observation-carried-forward (LOCF) method was used for imputation of missing data, and the *p* value was determined based upon a 2-sample *t-*test.

Secondary efficacy variables included mean IOP and mean IOP change from baseline at all follow-up visits. A mixed-model repeated measures (MMRM) analysis on observed values with unstructured covariance was performed with treatment, systemic *β*-blocker use (yes/no), time, and a treatment by time interaction term as factors, as well as subject as random effect. These variables were also analyzed using 2-sample *t*-tests with the LOCF method.

To further elucidate the IOP-lowering effects of both agents, post hoc responder analyses examining the percent reduction from baseline in IOP and mean IOP levels achieved were performed using observed data. Because of the numerically higher baseline IOP (0.5 mmHg) in the DFC group, a post hoc analysis of the mean IOP change from baseline was also conducted using an analysis of covariance (ANCOVA) model, with treatment as a fixed effect and baseline IOP as the covariate. In addition, the mean IOP change from baseline was analyzed for each baseline IOP subgroup (i.e., mean IOP < 25 and ≥25 mmHg, based on results from the Ocular Hypertension Treatment Study showing that patients with IOP < 25 mmHg were less likely to experience disease progression than those with IOP > 25 mmHg [43]) at all follow-up visits using 2-sample *t*-tests.

Safety assessments included AEs, visual acuity, biomicroscopy, ophthalmoscopy, cup/disc ratio, visual field, and vital signs. The safety population consisted of all patients who received at least one dose of study drug and attended at least one postbaseline visit. No imputation for missing data was performed.

All categorical variables were analyzed with Pearson's chi-square test or Fisher's exact test.

### 2.5. Sample Size Calculation

A sample size of 92 patients per group was determined based on the primary efficacy variable and the following assumptions (at week 12): a minimum difference of 1.5 mmHg in mean IOP change from baseline between treatment groups; standard deviation value of 3.4 mmHg (based on data from two pivotal studies of DFC, NCT00332384 and NCT00332436); 2-sided significance level of 0.05; 80% power; and 10% dropout rate (PASS 2000 software; NCSS LLC, Kaysville, UT, USA).

## 3. Results

### 3.1. Baseline Demographics and Patient Characteristics

A total of 192 patients were enrolled by eight centers (six in Mexico, two in Colombia) between June 2011 and September 2013. The safety and mITT populations included 191 and 190 patients, respectively. One patient was randomized but excluded from the safety and mITT populations after failing to attend any of the postbaseline visits. Another patient was excluded from the mITT population because no postbaseline efficacy data were available. Overall, 175/190 (92.1%) patients completed the study. Seven of the ninety-three (7.5%) patients in the TFC group withdrew from the study for the following reasons: AEs (*n* = 5; 5.4%), lost to follow-up (*n* = 1; 1.1%), and personal reasons (*n* = 1; 1.1%). Similarly, 8/97 (8.2%) patients in the DFC group withdrew for the following reasons: AEs (*n* = 4; 4.1%) and lost to follow-up (*n* = 4; 4.1%).

In the mITT population, demographics and baseline characteristics were similar between treatment groups (Table 1). All patients (100 from Mexico; 90 from Colombia) were Hispanic, and the majority required washout of their previous IOP-lowering medication (*n* = 56 [60.2%] in the TFC group; *n* = 62 [63.9%] in the DFC group). The PP population included 177 patients, of whom 168 (94.9%) completed the study; 5/87 (5.7%) and 4/90 (4.4%) patients in the TFC and DFC treatment groups, respectively, discontinued the study due to AEs. Demographics and baseline characteristics (including baseline mean IOP) of the PP population were similar to those of the mITT population, and no significant differences were observed between treatment groups (not shown).

### 3.2. Efficacy

In the mITT population, baseline mean IOP was comparable in the TFC (24.62 ± 2.48 mmHg) and DFC (25.12 ± 2.18 mmHg) groups (*p* = 0.139, 2-sample *t*-test). TFC provided statistically significantly greater mean IOP reduction at week 12 (primary efficacy variable) and all other postbaseline visits (2-sample *t*-tests), compared with DFC (Table 2). The between-treatment difference in mean IOP change from baseline favored TFC at all postbaseline visits (Table 2), and results were similar in the MMRM analysis.

**Figure 1:**
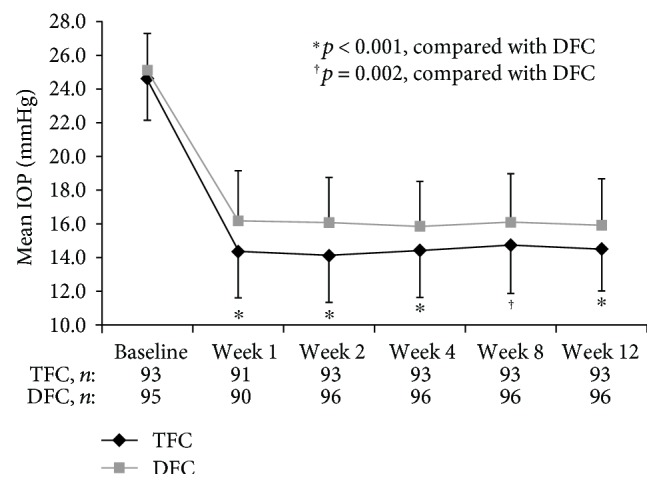
Mean intraocular pressure (IOP) at each visit in the modified intent-to-treat population. Data are presented as mean + standard deviation (SD) for TFC and mean − SD for DFC. Statistical significance was determined using the 2-sample *t*-test. DFC: dual fixed-combination; TFC: triple fixed-combination.

In both the mITT (Figure 1) and PP populations, mean IOP was statistically significantly lower at each postbaseline visit with TFC, compared with DFC (2-sample *t*-test). The between-treatment difference ranged from −1.91 to −1.33 mmHg in the mITT population and from −1.90 to −1.04 mmHg in the PP population (2-sample *t*-test), and similar results were obtained in both populations when analyzed with MMRM (e.g., −1.89 to −1.28 mmHg in the mITT population). In the responder analysis of % IOP change from baseline at week 12, a significantly greater proportion of patients achieved ≥40% IOP reduction from baseline in the TFC group (54.7%) than in the DFC (34.9%) group (*p* = 0.014). Additionally, although not reaching statistical significance, twice as many patients receiving TFC achieved ≥50% IOP reduction from baseline (15.1%) than with DFC (7.0%; Table 3). When looking at specific mean IOP achieved, a significantly greater proportion of patients achieved an IOP ≤ 13 mmHg with TFC than DFC (33.7% versus 14.8%, resp.; *p* = 0.004) and an IOP of ≤12 mmHg with TFC as well (16.3% versus 4.5%, resp.; *p* = 0.013).

Adjusting for the numerically higher baseline IOP in the DFC group (as described in Methods), the post hoc analysis (ANCOVA) of between-treatment differences revealed a greater mean IOP change from baseline with TFC in both the mITT (−1.08 mmHg; *p* ≤ 0.002) and the PP (−0.75 mmHg; *p* ≤ 0.046) populations at week 12 than with DFC. Statistical significance favoring TFC was also observed at all other postbaseline visits (weeks 1, 2, 4, and 8) in the mITT (*p* ≤ 0.005) and PP (*p* ≤ 0.021) populations.

**Figure 2:**
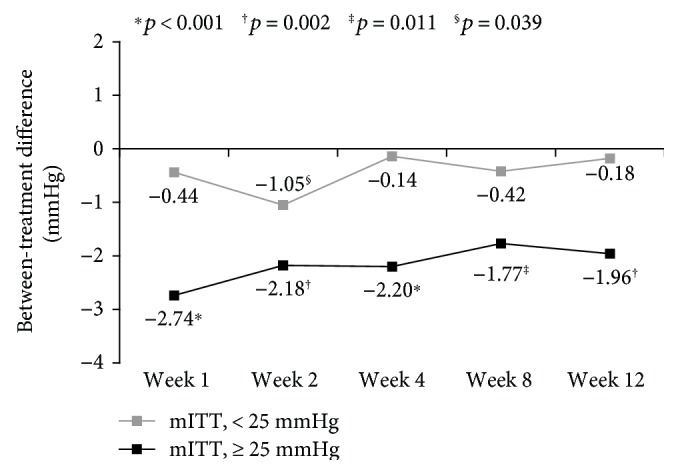
Between-treatment differences in mean intraocular pressure (IOP) change from baseline in patients with baseline IOP ≥ 25 versus <25 mmHg. Difference = TFC − DFC. Statistical significance was determined using the 2-sample *t*-test. Number of TFC and DFC patients, respectively, in each subpopulation: mITT, < 25 mmHg: 57, 51; mITT, ≥ 25 mmHg: 36, 46. DFC: dual fixed-combination; mITT,: modified intent-to-treat; TFC: triple fixed-combination.

In the subpopulation of patients with baseline mean IOP ≥ 25 mmHg, the between-treatment difference in mean IOP change from baseline statistically favored TFC at all postbaseline visits in both the mITT (*p* ≤ 0.011; Figure 2) and PP (*p* ≤ 0.032) populations. In contrast, there was no statistically significant difference in mean IOP change from baseline in the subpopulation of patients with baseline IOP < 25 mmHg, except at week 2 in the mITT population (*p* = 0.039; Figure 2).

### 3.3. Safety

In this 12-week study, a greater percentage of patients experienced one or more AEs with TFC (57/93; 61.3%) than DFC (39/98; 39.8%; *p* = 0.003); however, none were unexpected based on the components of TFC. Treatment-related AEs were reported in 49/93 (52.7%) patients receiving TFC and 27/98 (27.6%; *p* < 0.001) patients receiving DFC, of whom 45 (48.4%) and 15 (15.3%; *p* < 0.001) had AEs of an ocular nature, respectively. There were no serious ocular AEs. Four serious systemic AEs (bradycardia, pneumothorax, pyomyositis, and pituitary tumor) were reported in three patients in the TFC group, but none were considered treatment-related. Notably, the percentage of discontinuations due to AEs was similar in the TFC (5.4%) and DFC (4.1%; *p* = 0.742) groups. The most frequently reported AEs leading to discontinuation of TFC were conjunctival hyperemia (*n* = 3; 3.2%) and eye irritation (*n* = 2; 2.2%); allergic blepharitis, allergic conjunctivitis, dyspnea, and herpetic keratitis (*n* = 1; 1% each) led to discontinuation of DFC.

Treatment-related AEs reported in at least two patients in either treatment group are presented in Table 4. Although the percentage of patients reporting conjunctival hyperemia, eye irritation, or dry eye was statistically significantly greater with TFC than DFC (*p* ≤ 0.016), most reports of conjunctival hyperemia in the TFC group were mild to moderate in severity and only one was severe. In the DFC group, all reports of treatment-related conjunctival hyperemia were mild. Twice as many patients on DFC reported somnolence, although the difference did not reach statistical significance (Table 4).

At week 12, no clinically meaningful changes from baseline or statistically significant differences between groups were found in visual acuity, cup/disc ratio, ophthalmoscopy (except for one patient in the DFC group with signs of retinal vein occlusion), visual field, and vital signs. In an analysis of ≥2 severity grade increase from baseline in biomicroscopic findings, only conjunctival hyperemia was observed in a statistically significantly greater proportion of patients receiving TFC (12.9%), compared with DFC (2.0%; *p* = 0.004).

## 4. Discussion

In this multicenter, double-masked, randomized, phase 3 study, the primary efficacy analysis demonstrated that the triple fixed-combination bimatoprost 0.01%/brimonidine 0.15%/timolol 0.5% ophthalmic solution is superior to dual fixed-combination brimonidine 0.2%/timolol 0.5% in lowering IOP in patients with POAG and OHT. These findings are consistent with the expectation that the addition of bimatoprost would complement and augment the IOP-lowering effects of brimonidine and timolol.

In the mITT population at week 12, TFC produced a statistically significantly greater reduction in mean IOP from baseline than DFC. Sensitivity analyses in the PP population supported the results of the primary analysis, as did ANCOVA models that adjusted for baseline IOP in both the mITT and PP populations. These findings support available information regarding triple fixed-combination therapies [44–46].

Further evidence of the statistical and clinical superiority of TFC (compared with DFC) was provided by the subpopulation analysis based on patients' baseline IOP (following washout of previous medications). In patients with baseline IOP ≥ 25 mmHg, the between-treatment difference in mean IOP change from baseline statistically significantly favored TFC at all postbaseline visits in both the mITT and PP populations. This finding is clinically relevant as several landmark clinical trials have established the correlation between elevated IOP and increased risk of disease progression [43, 47–50], demonstrating that patients with elevated IOP, especially ≥25 mmHg, are at increased risk of disease progression, and that lowering IOP can lower this risk. These higher-risk patients may thus benefit disproportionately from therapies such as TFC that could both simplify their treatment regimen and offer the best opportunity to achieve the required IOP reduction.

Consistent with the hypothesis that patients with baseline IOP ≥ 25 mmHg are likely to require more medications to achieve a low, target IOP, the subpopulation of patients with baseline IOP < 25 mmHg exhibited similar IOP reductions with TFC and DFC treatments, possibly due to a floor effect (i.e., in the lower baseline IOP subpopulation, the treatment effect is not likely to be large enough to demonstrate a statistical difference between treatment groups) [51].

Additional evidence of the superior IOP-lowering efficacy of TFC is supplied by the responder analyses. More robust % decreases in IOP were observed with the TFC versus the DFC. Although it did not reach statistical significance, more than twice as many patients experienced a decrease in IOP of ≥50% with the TFC than the DFC. For patients requiring multiple medications and substantial IOP lowering, this difference may hold important clinical significance. Supportive of this important difference between groups is the fact that a substantially greater proportion of patients showed a response of ≥40% change from baseline in IOP in response to the TFC. Another very robust finding in the responder analysis was the statistically significant difference in patients achieving the very low, predetermined levels of IOP shown in Table 3. Recall that in the associative analysis of the landmark AGIS trial [49], patients whose IOP was <18 mmHg at 100% of visits had a mean IOP of 12.3 mmHg, and their mean change from baseline in visual field defect score was close to zero. In the current study, the results of the mean IOP responder analysis showed that substantially more patients in the TFC group reached the very low IOP levels of ≤13, and even ≤12 mmHg, and the results were highly significant.

TFC did not increase safety concerns, compared with DFC: no unexpected AEs were recorded and the discontinuation rate due to AEs was comparable; no clinically significant differences were observed between groups when visual acuity, cup/disc ratio, and visual field were evaluated, and the tolerability profile of TFC was consistent with that of its individual components. The greater proportion of patients with treatment-related AEs in the TFC group was indeed expected, as was the most common treatment-related AE, conjunctival hyperemia, in line with the use of bimatoprost 0.01% monotherapy [10]. In this study design, a conservative approach to analyzing AEs was taken, and findings were not predefined as requiring a specific grade change from baseline or severity level to be included in the AE analysis. This may have led to a proportion of conjunctival hyperemia in the TFC group being considered AEs for analysis purposes that were not of clinical relevance to patients. Regardless, all cases of conjunctival hyperemia but one were mild to moderate in the TFC group. In a commonly used analysis of a 2-grade or greater change from baseline severity, the incidence of hyperemia on biomicroscopic examination in the TFC group was only 12.9% (versus 2% for DFC). This is consistent with the discontinuation rate between groups being similar. It is also noteworthy that the incidence of somnolence was numerically lower with TFC, perhaps a result of the lower concentration of brimonidine (0.15%), compared with DFC (0.2%).

Bimatoprost is commonly known as a once-daily drug. It is important to point out, however, that differences between the formulation of TFC and the once-daily formulations (particularly the concentration of benzalkonium chloride (BAK)) allow bimatoprost to be administered twice daily as a component of TFC, while maintaining IOP-lowering efficacy consistent with the once-daily formulations. The originally marketed formulation of bimatoprost 0.03% contained 50 ppm BAK and was optimally administered once daily. The currently marketed formulation contains a lower concentration of bimatoprost (0.01%) in a higher concentration of BAK (200 ppm) and is also administered once daily. This suggests that the increased BAK concentration helps to enhance the penetration of bimatoprost into the eye to maintain efficacy consistent with the original bimatoprost 0.03% once-daily formulation [52]. TFC contains the lower concentration of bimatoprost (0.01%) in the lower concentration of BAK (50 ppm). Based on the previous studies, this difference was expected to allow TFC to be administered twice daily and maintain IOP-lowering effects and tolerability similar to the once-daily formulation [53–58]. It is also noteworthy that in a randomized, investigator-masked study of patients who instilled preservative-free bimatoprost 0.01% once daily for 3 weeks and increased their dosage to twice daily, there was no clinically meaningful or statistically significant difference in IOP lowering or tolerability between the once-daily and twice-daily dosing [53].

Other studies assessing the IOP-lowering efficacy and safety of TFC versus DFC have been performed in different patient populations [46, 59]; this is the first publication of the IOP-lowering efficacy and safety of the TFC. Future clinical studies assessing the long-term efficacy and safety of TFC are warranted to complement the results of this study. In addition, limiting enrollment to patients with baseline IOP ≥25 mmHg may be optimal since, as stated above, these higher-risk patients will benefit most from therapies such as TFC.

## 5. Conclusions

In patients with POAG or OHT and elevated IOP, TFC had superior ocular hypotensive effects (compared with DFC) while maintaining an acceptable safety/tolerability profile. TFC offers IOP-lowering benefits that may outweigh the risk of mostly mild ocular side effects for many patients. The advantages afforded by use of the TFC may be of particular clinical relevance for patients with higher baseline IOP who may be at increased risk of disease progression and thus need greater IOP lowering.

## Figures and Tables

**Table 1:** Baseline demographics and patient characteristics (modified intent-to-treat population).

Characteristics	TFC (*n* = 93)	DFC (*n* = 97)	*p* value^a^
Mean age (SD), years	60.0 (10.2)	58.8 (10.5)	0.411
≤65 years, *n* (%)	66 (71.0)	74 (76.3)	
>65 years, *n* (%)	27 (29.0)	23 (23.7)	
Gender, *n* (%)			0.151
Male	16 (17.2)	25 (25.8)	
Female	77 (82.8)	72 (74.2)	
Diagnosis, *n* (%)			0.505
OHT	32 (34.4)	29 (29.9)	
POAG	61 (65.6)	68 (70.1)	
Mean baseline IOP, mmHg (SD)	24.6 (2.5)	25.1 (2.2)	0.139
<25, *n* (%)	57 (61.3)	51 (52.6)	
≥25, *n* (%)	36 (38.7)	46 (47.4)	
Concurrent use of systemic *β*-blockers, *n* (%)			0.925
Yes	9 (9.7)	9 (9.3)	
No	84 (90.3)	88 (90.7)	

DFC: dual fixed-combination; IOP: intraocular pressure; OHT: ocular hypertension; POAG: primary open-angle glaucoma; SD: standard deviation; TFC: triple fixed-combination. ^a^Pearson's chi-square test.

**Table 2:** Mean IOP change from baseline in the modified intent-to-treat population (2-sample *t*-test).

Visit	Mean IOP change from baseline (SD), mmHg		Between-treatment difference^a^ (95% CI), mmHg	*p* value
	TFC	DFC		
Week 1	−10.18 (3.15)	−8.90 (3.04)	−1.28 (−2.19, −0.37)	0.006
*n*	91	89		
Week 2	−10.44 (2.90)	−9.04 (2.91)	−1.40 (−2.24, −0.55)	0.001
*n*	93	94		
Week 4	−10.14 (2.97)	−9.23 (2.27)	−0.92 (−1.68, −0.15)	0.019
*n*	93	94		
Week 8	−9.84 (3.00)	−8.99 (2.83)	−0.85 (−1.69, −0.01)	0.047
*n*	93	94		
Week 12^b^	−10.03 (2.66)	−9.18 (2.57)	−0.85 (−1.60, −0.09)	0.028
*n*	93	94		

CI: confidence interval; DFC: dual fixed-combination; IOP: intraocular pressure; SD: standard deviation; TFC: triple fixed-combination. ^a^Negative values indicate greater IOP lowering with TFC than DFC. ^b^Primary efficacy variable.

**Table 3:** Responder analyses: percentage reduction in IOP from baseline and mean IOP levels achieved^a^.

	Patients, *n* (%)		*p* value
	TFC	DFC	
IOP reduction at week 12			
≥40%	[*n* = 86]	[*n* = 86]	
	47 (54.7)	30 (34.9)	0.014
≥50%	[*n* = 86]	[*n* = 86]	
	13 (15.1)	6 (7.0)	0.143
IOP level at week 12			
≤13 mmHg	[*n* = 86]	[*n* = 88]	
	29 (33.7)	13 (14.8)	0.004
≤12 mmHg	[*n* = 86]	[*n* = 88]	
	14 (16.3)	4 (4.5)	0.013

DFC: dual fixed-combination; IOP: intraocular pressure; TFC: triple fixed-combination. ^a^Performed post hoc.

**Table 4:** Treatment-related adverse events reported in two or more patients in one treatment group.

Adverse event	TFC, *n* (%)	DFC, *n* (%)	*p* value^a^
	(*N* = 93)	(*N* = 98)	
Conjunctival hyperemia	22 (23.7)	3 (3.1)	<0.001^a^
Eye irritation	13 (14.0)	4 (4.1)	0.016^a^
Dry eye	8 (8.6)	1 (1.0)	0.016^b^
Eye pruritus	6 (6.5)	2 (2.0)	0.161^b^
Somnolence	4 (4.3)	8 (8.2)	0.272^a^
Allergic conjunctivitis	4 (4.3)	1 (1.0)	0.202^b^
Punctate keratitis	1 (1.1)	4 (4.1)	0.369^b^
Skin hyperpigmentation	3 (3.2)	0	0.114^b^
Asthenia	2 (2.2)	1 (1.0)	0.613^b^
Dizziness	2 (2.2)	1 (1.0)	0.613^b^
Eyelid pruritus	2 (2.2)	0	0.236^b^
Dry mouth	0	2 (2.0)	0.498^b^

DFC: dual fixed-combination; TFC: triple fixed-combination. ^a^Pearson's chi-square test. ^b^Fisher's exact test.
